# Enhancing prognostic accuracy in head and neck squamous cell carcinoma chemotherapy via a lipid metabolism-related clustered polygenic model

**DOI:** 10.1186/s12935-023-03014-5

**Published:** 2023-08-11

**Authors:** Xiangwan Miao, Hao Wang, Cui Fan, QianQian Song, Rui Ding, Jichang Wu, Haixia Hu, Kaili Chen, Peilin Ji, Qing Wen, Minmin Shi, Bin Ye, Da Fu, Mingliang Xiang

**Affiliations:** 1 Department of Otolaryngology & Head and Neck Surgery, Ruijin Hospital, Shanghai Jiao Tong University School of Medicine, Shanghai, China; 2 Shanghai Key Laboratory of Translational Medicine on Ear and Nose Diseases, Shanghai, China; 3 Ear Institute, Shanghai Jiao Tong University School of Medicine, Shanghai, China; 4 Department of Otorhinolaryngology, Ruijin Hospital Lu Wan Branch, Shanghai Jiao Tong University School of Medicine, Shanghai, China; 5 Department of Cancer Biology, Wake Forest University School of Medicine, Winston-Salem, USA; 6 Research Institute of Pancreatic Diseases, Shanghai Jiao Tong University School of Medicine, Shanghai, China; 7 State Key Laboratory of Oncogenes and Related Genes, Shanghai, China; 8 Institute of Translational Medicine, Shanghai Jiao Tong University, Shanghai, China

**Keywords:** HNSCCs, Prognostic model, Chemotherapy, Precision medicine, Lipid metabolism

## Abstract

**Objective:**

Systemic chemotherapy is the first-line therapeutic option for head and neck squamous cell carcinoma (HNSCC), but it often fails. This study aimed to develop an effective prognostic model for evaluating the therapeutic effects of systemic chemotherapy.

**Methods:**

This study utilized CRISPR/cas9 whole gene loss-of-function library screening and data from The Cancer Genome Atlas (TCGA) HNSCC patients who have undergone systemic therapy to examine differentially expressed genes (DEGs). A lipid metabolism-related clustered polygenic model called the lipid metabolism related score (LMRS) model was established based on the identified functionally enriched DEGs. The prediction efficiency of the model for survival outcome, chemotherapy, and immunotherapy response was evaluated using HNSCC datasets, the GEO database and clinical samples.

**Results:**

Screening results from the study demonstrated that genes those were differentially expressed were highly associated with lipid metabolism-related pathways, and patients receiving systemic therapy had significantly different prognoses based on lipid metabolism gene characteristics. The LMRS model, consisting of eight lipid metabolism-related genes, outperformed each lipid metabolism gene-based model in predicting outcome and drug response. Further validation of the LMRS model in HNSCCs confirmed its prognostic value.

**Conclusion:**

In conclusion, the LMRS polygenic prognostic model is helpful to assess outcome and drug response for HNSCCs and could assist in the timely selection of the appropriate treatment for HNSCC patients. This study provides important insights for improving systemic chemotherapy and enhancing patient outcomes.

**Supplementary Information:**

The online version contains supplementary material available at 10.1186/s12935-023-03014-5.

## Background

Squamous cell carcinomas of the head and neck (HNSCCs) have a devastating impact on individuals worldwide. HNSCCs affect 850,000 people annually and result in 440,000 deaths [1, 2]. Unfortunately, over 40% of HNSCC patients are diagnosed at an advanced stage due to a lack of specific symptoms in early stages [3]. Patients in stages III and IV may have extensive local tumor invasion, and only 30% may survive for five years [4]. The treatment of HNSCCs varies according to the pathological features and disease stage and can include surgery or concurrent chemoradiotherapy and immunotherapy [3, 5–7]. However, extensive tissue excision and the toxic side effects of chemo- and radiotherapy significantly harm the quality of life and overall outcome of HNSCC due to swallowing and respiratory dysfunction [8].

The advent of immune checkpoint inhibitors has resulted in a significant improvement in the overall survival of HNSCCs [9, 10]. However, only a limited population could benefit from this approach. Cisplatin-based chemotherapy has a wider range of applications and can be used in more populations than immunotherapy. Moreover, patients who are sensitive to cisplatin-based systematic therapy have better overall survival and quality of life, and tumor downstaging and opportunities to protect swallowing and speech functions may be possible [6]. However, most HNSCC patients are still diagnosed in advanced stages, and treatment failures still occur [7], even with personalized comprehensive treatment. Despite the improvement in treatment for locally advanced HNSCC, recurrences are still observed in nearly half of cases [8]. Thus, reliable options for platinum-resistant diseases are needed [9]. This is critical, as only a few targeted drugs, including programmed death-1 (PD-1) inhibitors, programmed death-ligand 1 (PD-L1) inhibitors, and cetuximab ^7^, are available. Hence, it is essential to identify potential drug resistance molecules and mechanisms to develop novel targeted drugs and formulate comprehensive treatment plans.

The metabolism of lipids is vital for the processes underlying cancer development and progression [10] and drug resistance [11–13]. Recent studies have demonstrated that alterations in the expression of genes involved in lipid metabolism are associated with cisplatin resistance, particularly those engaged in the metabolism of fatty acids, such as FASN [14], CD36 [20] and SCD [16]. Abnormal lipid metabolism in HNSCC includes enhanced de novo lipid synthesis or accumulation and/or impaired lipolysis [17]. According to previous studies, there is a strong association between elevated CPT1A-dependent lipolysis and resistance to radiotherapy in individuals diagnosed with nasopharyngeal carcinoma [18]. Additionally, radiotherapy resistance is closely related to fatty acid synthesis in HNSCCs [19]. However, the occurrence of reprogramming of lipid metabolism is specific to therapeutic drugs and is affected by the environment and cellular microenvironment [20, 21]. Previous studies on lipid metabolic reprogramming in HNSCCs are limited, and conflicting results have been reported [17] based on different anatomical regions and tumor microenvironments.

**Fig. 1:**
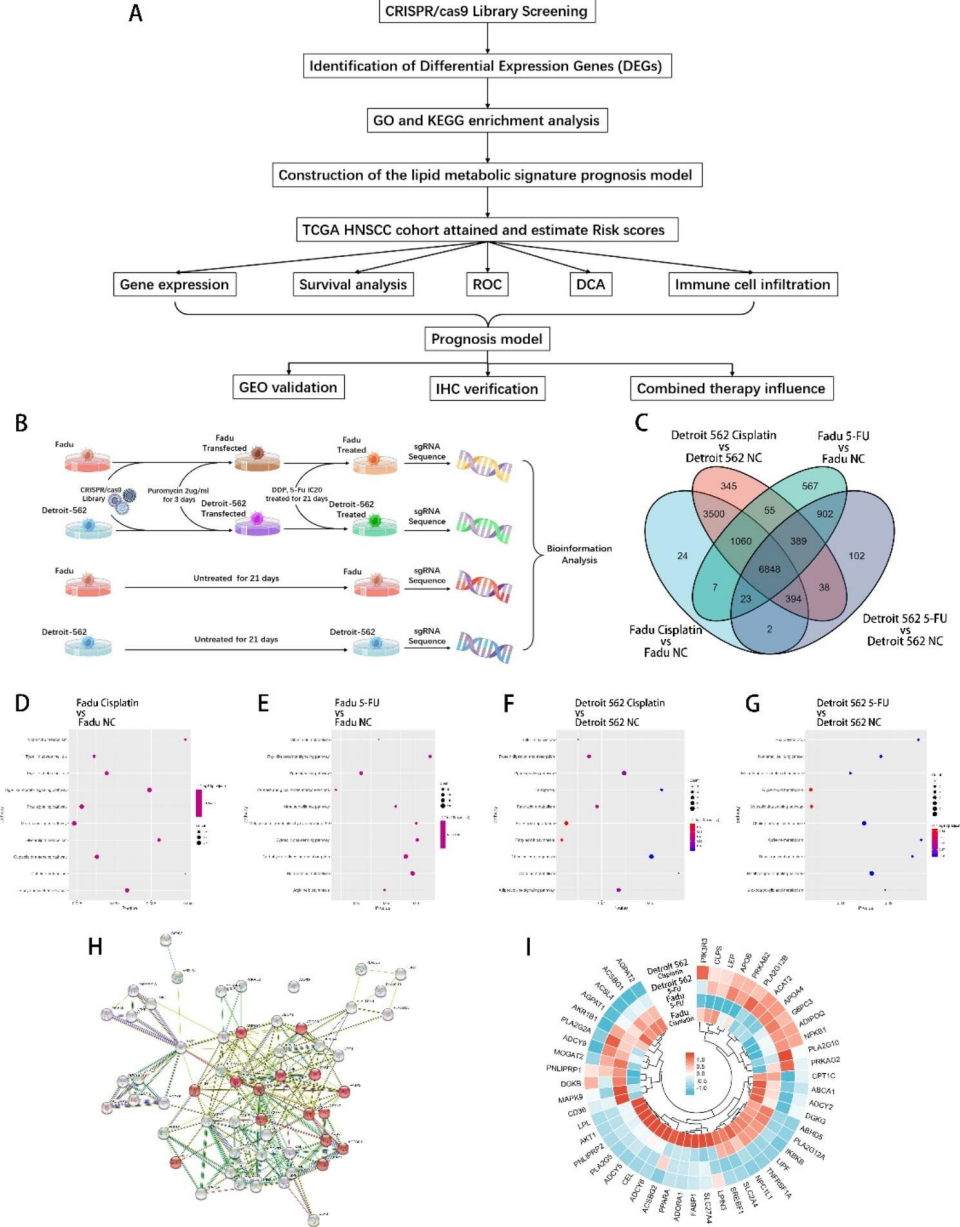
**Lipid metabolism-related genes were significantly enriched in CRISPR/cas9 library screening. A**, Illustration of the stepwise study design. **B**, Illustration of CRISPR/cas9 library screening in the HNSCC cell lines Fadu and Detroit 562 cells. **C**, Venn diagrams showing the 6,848 candidate genes related to drug resistance. **D-G**, GO and KEGG enrichment analyses for cisplatin and 5-FU drug screening in Fadu and Detroit 562 cells. **H**, PPI network analysis of the screening for differentially expressed lipid metabolism genes. **I**, Heatmap for differentially expressed genes involved in lipid metabolism across the four screening groups

The objective of this research was to investigate the influence of lipid metabolism reprogramming on the overall drug response, particularly that of chemotherapy, in HNSCCs. Here, we integrated CRSIPR/cas9 whole gene loss-of-function library screening and the TCGA HNSCC dataset and identified that cisplatin resistance is associated with genes related to lipid metabolism. We constructed a polygenic model for HNSCC prognostic prediction, called the LMRS model, that is based on eight clustered lipid metabolism genes. Additionally, we evaluated the effectiveness and prognostic value of the LMRS model for overall outcome, drug response, and immunotherapy response prediction in HNSCCs. Validation was performed not only in TCGA HNSCC and GEO datasets but also on collected clinical specimens. Finally, we verified that the constructed LMRS model achieved good prognosis prediction and provided an essential reference for diagnostic and therapeutic decisions in HNSCC patients (Fig. 1A). Our findings highlight the significance of lipid metabolism in drug response and provide potential drug targets and improved prognostic models.

## Methods and materials

### Cell culture and cell viability assay

Fadu and Detroit-562 cells were purchased from the Cell Bank of the Chinese Academy of Sciences and kept in a cell incubator with 5% CO_2_ at 37 °C in high-glucose DMEM (Gibco, Grand Island, NY, USA) containing L-glutamine with 10% fetal bovine serum, penicillin (100 U/mL), and streptomycin (100 U/mL). The CCK-8 assay was used to assess cell viability, proliferation, and drug resistance. Drugs were added 24 h after the cells in an exponential growth phase were plated in 96-well plates to test cell viability. The cells were treated with 10 µL of CCK-8 (MedChemExpress, Monmouth Junction, NJ, USA) according to the manufacturer’s instructions. After 2 h, the absorbance was measured at 450 nm. Three parallel experiments were carried out for each sample, and at least five replicate wells were used to analyze each concentration. Rosiglitazone and Orlistat were purchased from MCE (MedChemExpress, Monmouth Junction, NJ, USA). Annexin V-Propidium Iodide kit was used to determine the apoptosis cells distribution following the manufacturer’s instructions (Solarbio, Beijing, China).

### Quantitative RT‒PCR, cDNA synthesis, and RNA isolation

Total RNA was isolated from cell lines using TRIzol according to the manufacturer’s instructions (TaKaRa, Dalian, Liaoning, China). The PrimeScript RT reagent kit for cDNA synthesis was used for reverse transcription experiments (TaKaRa). The RNA concentration was determined by measuring the absorbance at 260 nm. After normalizing the expression to that of beta-actin, the expression values were calculated relative to those of the control samples.

### CRISPR/cas9 loss-of-function library screening

CRISPR/cas9 loss-of-function library screening was employed using the protocol outlined in Nature protocols [22]. Lentiviral products were obtained from OBiO Technology (Shanghai) Corp., Ltd. (Shanghai, China). Screening was performed in both Fadu and Detriot-562 cells, which were generated from human primary hypopharyngeal carcinoma and human lung metastasis of hypopharyngeal cancer, to identify genes whose suppression desensitized cancer cells to chemotherapy. The cell lines were plated in 100-mm plates at 500,000 cells per well one day before the experiment. The cells were starved for 12 h and then transduced at an MOI of 0.3, 0.4, and 0.5. After 18 h, the culture supernatant was removed, and the transduced cells were kept in complete culture medium with 2 µg/mL puromycin for more than 3 days to generate knockout cells (KO cells). The KO cells were divided into normal and treated groups using three duplicates of cells under different chemical treatments: DDP (5 µg/mL for Detroit-562 and 1 µg/mL for Fadu) and 5-FU (5 µg/mL for Detroit-562 and 1 µg/mL for Fadu) at an IC20 dosage. Every 3–5 days, fresh high-glucose DMEM that contained toxins was changed. After a two-week selection, sgRNA PCR amplification and next generation sequencing of the extracted DNA from the different groups were performed.

Using MAGeCKO (version 0.5.7), sgRNA labels were matched to the human reference (hg19). Using robust rank aggregation analysis, differential analysis of the screened sgRNAs between DDP- or 5-FU-treated and control groups was carried out. Based on the following criteria, sgRNA enrichment was selected for additional analysis: sgRNAs with an FDR of 0.25 and p value of 0.05 for each independent replication in the cisplatin and 5-FU groups or sgRNAs with either an FDR of 0.05 or p value of 0.05 for each independent replication in the DDP and 5-FU groups. Azenta Life Sciences (Shanghai, China) assisted in CRISPR/cas9 screening and sgRNA data analysis.

### GO function and KEGG pathway enrichment analyses of the differentially expressed sgRNAs

The R environment cluster profile (version 3.14) and package “org.Hs.eg.db (version 3.10)” were used for GO function and KEGG pathway enrichment analyses of the differentially expressed sgRNAs from the CRISPR/cas9 library screening. Differential analysis of biological processes (BPs), cellular components (CCs), molecular functions (MFs), and related pathways was performed. The BH method of multiple testing correction was used.

### TCGA and GEO datasets

Clinical information, TCGA RNA-seq data, and probe annotation files were collected from the TCGA HNSCC dataset (https://portal.gdc.com). Samples without clinical information were disregarded. Using the R tool “GEOquery,” the GSE32877, GSE10300, and GSE41613 datasets were obtained from the Gene Expression Omnibus (GEO) database.

### Establishment of the LMRS model

Gene counts from the TCGA HNSCC patients who had received systematic therapy were converted to TPM. The data were normalized using log2 (TPM + 1), and only samples with available clinical data were analyzed. There were 173 samples included in further analyses.

#### Subgroup division

The data were analyzed over 100 times with the R package “ConsensusClusterPlus (version 1.54)”, with clusterAlg = “hc” and innerLinkage = “ward. D2” to cluster two subgroups. The R package “pheatmap” (version 1.0.12) was then used to construct the heatmaps, and only gene expression with SD > 0.1 was included. If the input gene count exceeded 1,000, the top 25% of genes were extracted, and the SD was filtered. The prcomp function in the R environment was used to perform PCA.

#### Establishment of the 8-gene signature LMRS model

There were 751 lipid metabolism-related genes shared between the two groups. Integrated with the CRISPR/cas9 library screening results, the top 50 genes related to lipid metabolism were adopted. The package “glmnet” was used for the least absolute shrinkage and selection operator (LASSO) algorithm as well as the feature selection and 10-fold cross-validation. Based on the median score, the samples were divided into high and low groups. Each patient’s score was calculated using a formula weighted by their regression coefficient from the above analysis.

Score = (− 2.7486) * ACSBG2 + (1.7158) * APOB + (− 0.3216) * IKBKB + (0.4612) * MAPK9 + (− 0.8421) * MOGAT2 + (0.6413) * PLA2G10 + (− 0.2157) * PIK3R3 + (− 0.2355) * SREBF1.

### Analysis of prognosis

For Kaplan‒Meier curves, log-rank tests were used to assess p values and hazard ratios (HRs). To select the appropriate terms, univariate and multivariate Cox regression analyses were used for the nomogram. The forest plot was used to display the p value, HR, and 95% CI of each variable using the package “forestplot”. Then, using the R package “rms”, a nomogram was developed based on the results of the multivariate Cox proportional hazards analysis to predict the 1-, 3-, and 5-year overall recurrence rates.

To compare the predictive power of the established lipid metabolism subgroups, the 8-gene LMRS model, individual genes in the LMRS model, and other clinical risk factors, the package “timeROC (version 0.4)” was used. Prediction accuracy was measured using a time-dependent receiver operating characteristic (ROC) curve and area under the curve (AUC).

The decision curve analysis R package “ggDCA” was applied to generate diagnostic models for 1-, 3-, and 5-year survival.

### Immune function analysis

We used the R package “immuneeconv” to assess the reliability of the results of the immune score. This package combines six of the most recent algorithms, including quanTIseq, CIBERSORT, xCell, MCP-counter, and TIMER. These algorithms have distinct advantages and have been benchmarked.

The immune checkpoint-related transcripts SIGLEC15, TIGIT, CD274, HAVCR2, PDCD1, CTLA4, LAG3, and PDCD1LG2 were selected. The expression profiles of these genes were obtained from the TCGA HNSCC cohort. The “TIDE” algorithm was used to forecast the potential immune checkpoint blockade (ICB) responses. Correlations between gene expression and immunological score were visualized using the R package “ggstatsplot”. Multigene correlation was visualized by the R package “pheatmap”.

### GEO dataset validation

The GEO datasets GSE32877 and GSE10300 were mined for the expression profiles of the LMRS genes, which were used to determine the prognosis scores. The relationship between the prognosis score to the outcomes of response to systematic therapy and survival outcomes was further examined.

### Sample collection and immunohistochemical and immunofluorescence staining analysis

Primary tissue samples (HNSCC, n = 30) and biochemical data were anonymized and obtained in accordance with the policies of the Ruijin Hospital (Shanghai, China) Institutional Review Board. After fixing in 4% paraformaldehyde, samples were cut into 4 μm-thick sections and embedded in paraffin blocks. Following hematoxylin-eosin (HE) staining, a second tumor slice was incubated overnight in SREBF1, PIK3R3, MAPK9, IKK2, APOB, ACSBG2, MOGAT2, and PLA2G10 antibodies (SREBF1/PIK3R3; Abcam, Boston, MA, U.S. A; MAPK9: CST, Boston, MA, U.S. A; APOB/IKBKB: Beyotime Biotech Inc., Shanghai, China; ACSBG2: BBI, Shanghai, China; MOGAT2: Bioss, Beijing, China) at 4 °C. The next day, the samples were treated with secondary antibodies at room temperature for two hours before imaging. Slices were imaged using the Zeiss Zen 3.3 system followed by ImageJ software.

### Statistical analyses

R software (version 4.0.3) and ggplot2 (version 3.3.2) were used for statistical analyses (R Foundation for Statistical Computing, Vienna, Austria). Spearman’s correlation method, which evaluates the correlation between nonnormally distributed numerical variables, was employed to investigate the relationships between the immunological score and gene expression. Statistical significance was defined as a p value of less than 0.05.

## Results

### Lipid metabolism genes associated with HNSCC chemotherapy resistance were identified via CRISPR/Cas9 library screening

The GeCKO 2.0 CRISPR/Cas9 library for loss-of-function screening covers 3–6 sgRNAs per gene and is based on whole-population mRNA. GeCKO 2.0 was transfected into the HNSCC cell lines Fadu and Detroit 562 for loss-of-function screening to understand the functions and pathways involved in chemotherapy resistance in HNSCCs (Fig. 1B). To select the appropriate concentration for drug screening, the IC20 concentrations of cisplatin and 5-FU were then used for drug screening (Supplementary Table S1). The IC20 values of cisplatin in Fadu and Detroit 562 cells were 2.5 and 1.25 µg/ml, respectively, while those of 5-FU were 6 and 0.07 µg/ml, respectively. Treated cells were then employed for next-generation sequencing to obtain the DEGs. The related drug resistance candidate genes were narrowed to 6,848 DEGs that were expressed in all comparison groups; of these DEGs, 731 had significant differences (639 positively screened genes and 92 negatively screened genes with a significance threshold of p < 0.05 and a false discovery rate (FDR) < 0.25) (Fig. 1C). The top-ranking genes included those that contribute to tumor development and drug resistance, such as AKT1, KLF6, and NCOR2. Furthermore, the results revealed several commonly upregulated genes in HNSCC, such as KRT5 and TGFBRAP1 (Supplementary Figure S1), confirming that the identified genes from screening were highly related to drug resistance in HNSCCs. Moreover, the results also confirmed that the knockout of some genes, such as BEX2, significantly decreased the survival and proliferation rates and induced death in HNSCC tumor cells.

GO functional enrichment and KEGG pathway analysis revealed a significant enrichment of genes related to lipid metabolism functions and pathways (Fig. 1D–G; Supplementary Figure S2), especially genes involved in the PPAR pathway and glycerin and fatty acid metabolism. The analysis of the protein‒protein interaction network for DEGs associated with lipid metabolism showed several clusters (Fig. 1H). Heatmaps of lipid-related DEGs showed that their expression slightly differed according to cell lines and drugs (Fig. 1I). Our findings imply that lipid metabolism reprogramming is a crucial factor in the drug response of HNSCCs as the combination of cisplatin and lipid metabolism regulation drugs lead to enhanced apoptosis in HNSCC cell lines (Supplementary Figure S3). Biochemical analysis of the clinical samples validated that the chemotherapy responder cohort had notably elevated serum triglyceride levels compared to the nonresponder group (Table 1). As triglycerides use fatty acids as one of their primary substrates, we further hypothesize that lipid metabolism-related genes may serve as prognostic markers for predicting the overall outcome and drug response of HNSCCs.

**Table 1:** Characteristics of clinical samples

Characteristic		Responder	Nonresponder	P Value
**Number, n**	10		20	
**Gender, n (%)**				**1.000**
Male	10 (33%)		20 (67%)	
Female	0 (0%)		0 (0%)	
**Smoke, n (%)**				**0.008**
No	4 (40%)		0 (0%)	
Yes	6 (60%)		20 (100%)	
**Alcohol, n (%)**				**0.045**
No	7 (70%)		5 (25%)	
Yes	3 (30%)		15 (75%)	
**Weight, median (IQR)**	75 (62, 75)		67 (65.6, 74)	**0.219**
**BMI, mean ± SD**	23.544 (21.765, 24.212)		23.128 (22.501, 24.102)	**0.947**
**Triglycerides, ** **mmol/L, mean ± SD**	2.25 (2.055, 2.6475)		1.05 (0.905, 1.4425)	**< 0.001**
**Free fatty acid, ** **mmol/L, mean ± SD**	0.399 ± 0.093506		0.43176 ± 0.13347	**0.502**
**T stage, n (%)**				**0.861**
T2	2 (20%)		4 (20%)	
T3	7 (70%)		11 (55%)	
T4	1 (10%)		5 (25%)	
**N stage, n (%)**				**0.563**
N0	1 (10%)		4 (20%)	
N1	4 (40%)		4 (20%)	
N2	5 (50%)		12 (60%)	
**M Stage, n (%)**				**1.000**
M0	10 (33%)		20 (67%)	
M1	0 (0%)		0 (0%)	

**Fig. 2:**
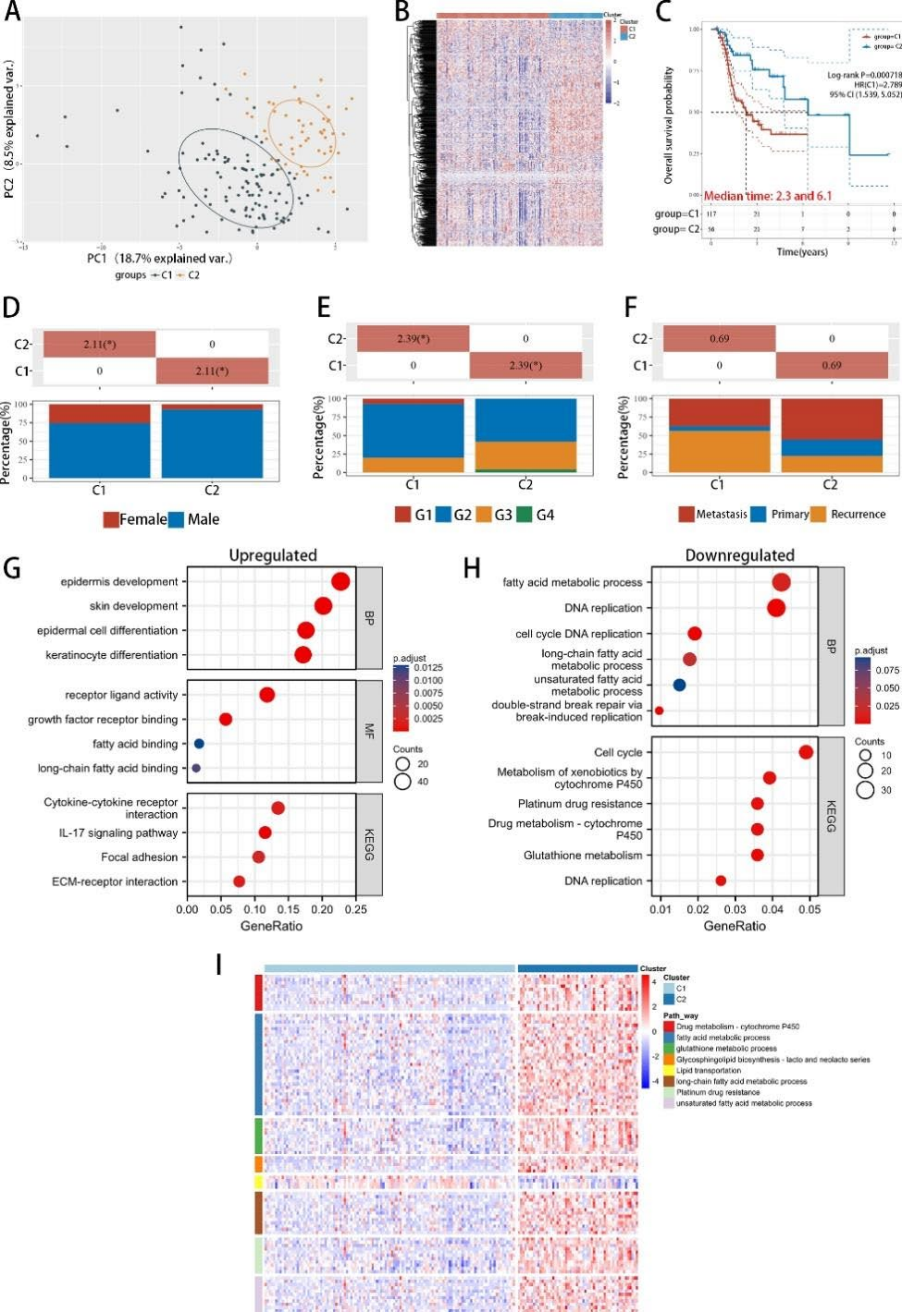
**The prognosis of HNSCC patients undergoing systemic therapy using lipid metabolism genes showed different outcomes and metabolic characteristics. A**, Principal component analysis revealed two subgroups in all 173 TCGA-HNSCCs samples. **B**, Heatmap of the lipid metabolism genes in the two subgroups. **C**, Survival curve of the two subgroups. **D**, Gender distribution between the C1 and C2 groups. **E**, Pathology grade distribution between the C1 and C2 groups. **F**, Tumor status distribution between the C1 and C2 groups. **G**, GO and KEGG enrichment analyses for upregulated DEGs. **H**, GO and KEGG enrichment analyses for downregulated DEGs. **I**, Heatmap of the expression of metabolism-related genes in the two subgroups

We next investigated the functions of all 751 known lipid metabolism genes in HNSCC. Patients from the TCGA-HNSCC cohort with a history of chemotherapy or targeted therapy (n = 173; Supplementary Table S2) were divided into two subtypes (group Cluster 1, C1, n = 117; group Cluster 2, C2, n = 56) according to the varying expression levels of the 751 genes related to lipid metabolism (Fig. 2A). The heatmap revealed noticeable variances in the lipid metabolism-associated gene expression patterns between the C1 and C2 groups (Fig. 2B). Survival analysis, combined with clinical data, showed that the C1 group had a significantly worse clinical prognosis (median survival: 2.3 years) than the C2 group (median survival: 6.1 years), with p values < 0.05 (Fig. 2C). Furthermore, the clinical characteristics also showed some differences between the groups. The C1 group showed a larger proportion of female patients than the C2 group (Fig. 2D). Moreover, the C1 group had a higher proportion of well- and moderately differentiated patients and a lower proportion of poorly and undifferentiated patients (Fig. 2E). The other clinical characteristics were not different between the two subgroups (Fig. 2F; Supplementary Figure S4A). Additionally, GO and KEGG analyses indicated that the C1 group had more genes enriched in keratinocyte differentiation and cytokine‒cytokine receptor interactions, whereas the C2 group had a greater number of genes enriched in fatty acid metabolism and DNA replication (Fig. 2G and H). The heatmap of metabolism-related genes demonstrated that genes related to drug metabolism, platinum drug resistance, glutathione metabolism, and fatty acid metabolism had relatively lower expression levels in the C1 group, whereas only those related to lipid transportation showed a relatively lower expression in the C2 group (Fig. 2I). These results support our hypothesis that lipid metabolism-related genes play vital roles in drug responses.

### A polygenic prognostic model with lipid metabolism characteristics was generated

**Fig. 3:**
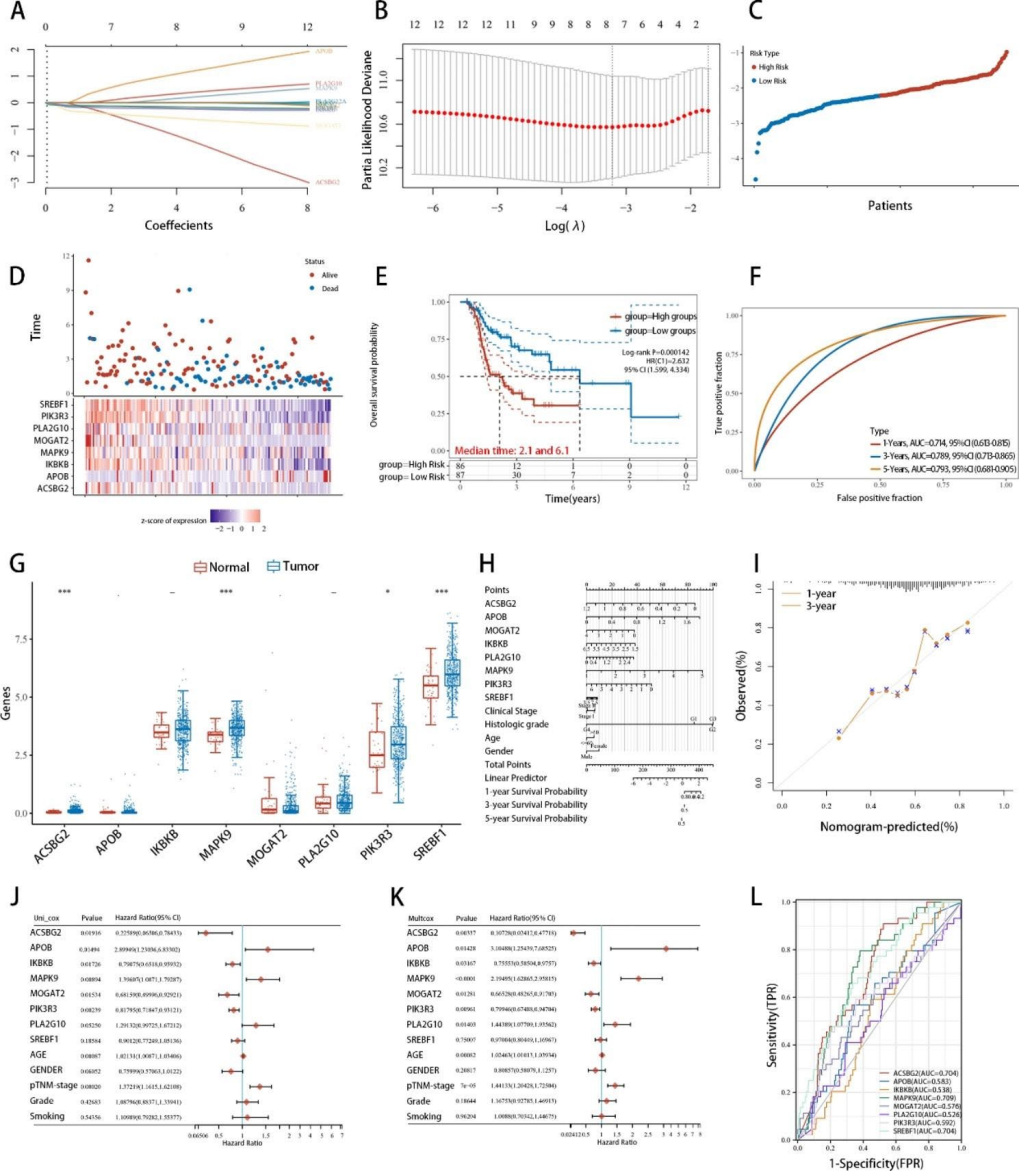
**The LMRS model was generated and predicted the prognosis of HNSCC. A-B**, The LMRS model was constructed using the eight signature genes found to be highly linked with overall survival (OS) in LASSO Cox regression. **C**, The scores of patients with TCGA-HNSCCs who received systematic therapy. **D**, Survival status of patients among TCGA-HNSCCs who received systematic therapy. **E**, Survival time of patients among TCGA-HNSCCs who received systematic therapy. **F**, The ROC curve and AUC for the LMRS model at 1, 3, and 5 years. **G**, The differences in LMRS model gene expression between normal and tumor groups in the TCGA HNSCC dataset. **H**, OS nomogram; **I**, OS nomogram model calibration curve. **J**, Forest plot of the univariate analysis for the LMRS model. **K**, Forest plot for multivariate analysis of the model. **L**, ROC curve for genes included in the LMRS model

A comprehensive testing panel encompassing 751 genes is not only time-consuming but also expensive. Hence, a succinct and efficient evaluation model can significantly diminish the consumption of resources in terms of time, funds, and human effort. Through LASSO regression analysis in conjunction with previous CRISPR/cas9 library screening findings, the number of genes incorporated in this assessment model was minimized. Feature selection was accomplished through the application of the least absolute shrinkage and selection operator regression (LASSO regression), supplemented with tenfold cross validation. Ultimately, the LMRS model comprises eight genes related to lipid metabolism (ACSBG2, APOB, IKBKB, MAPK9, MOGAT2, PLA2G10, PIK3R3, and SREBF1), as depicted in Fig. 3A, B. These genes are involved in various lipid metabolism processes and their regulatory pathways, particularly fatty acid metabolism since lipid metabolism regulation drugs significantly contribute to the expression of LMRS model genes (Supplementary Figure S3). The model calculation formula was:

Score = (− 2.7486) * ACSBG2 + (1.7158) * APOB + (− 0.3216) * IKBKB + (0.4612) * MAPK9 + (− 0.8421) * MOGAT2 + (0.6413) * PLA2G10 + (− 0.2157) * PIK3R3 + (− 0.2355) * SREBF1.

The scores were determined based on the LMRS gene expression levels. Then, they were separated into two groups, LMRS-High and LMRS-Low, based on their high and low LMRS scores, respectively (Fig. 3C, D). Notably, the LMRS-High group exhibited lower overall survival rates and increased death rates with a median survival of 2.1 years. The LMRS-Low group demonstrated higher overall survival rates, with a median survival of 6.1 years and a p value < 0.05 (Fig. 3E). These survival outcome results were also observed in subgroups categorized by the 751 lipid metabolism-related genes. The prognostic evaluation model based on the LMRS was diagnostically effective, as its area under the curve (AUC) metrics exceeded 0.7 at one, three, and five years (1 year = 0.714. 95% CI, 0.613–0.815; 3 year = 0.789. 95% CI, 0.713–0.865; 5 year = 0.793. 95% CI, 0.681–0.905.). Additionally, the model exhibited greater effectiveness over medium time frames relative to shorter ones (Fig. 3F).

### The LMRS model had an outperformed prognostic value in HNSCCs

Except for ACSBG2, MAPK9, PIK3R3, and SREBF1, the remaining genes in the LMRS model exhibited no significant expression variations in the whole TCGA-HNSCC patient cohort, which could be attributed to the diverse treatments undertaken by each patient. Additionally, most genes were upregulated in HNSCCs (as depicted in Fig. 3G). A nomogram and calibration curve were constructed using these eight genes and demonstrated good consistency and a c-index of 0.672 (0.651–0.692) (Fig. 3H, I). Univariate and multivariate regression results revealed the significance of these genes. ACSBG2, IKBKB, and PIK3R3 were protective factors, whereas APOB and MAPK9 were hazard factors in HNSCCs (Fig. 3J-K). However, MOGAT2, PLA2G10, and SREBF1 showed no significant differences. Regarding the efficacy of diagnosis, only ACSBG2, MAPK9 and SREBF1 exhibited AUCs greater than 0.7 (ACSBG2 AUC = 0.704; MAPK9 AUC = 0.709; SREBF1 AUC = 0.704) (Fig. 3L).

**Fig. 4:**
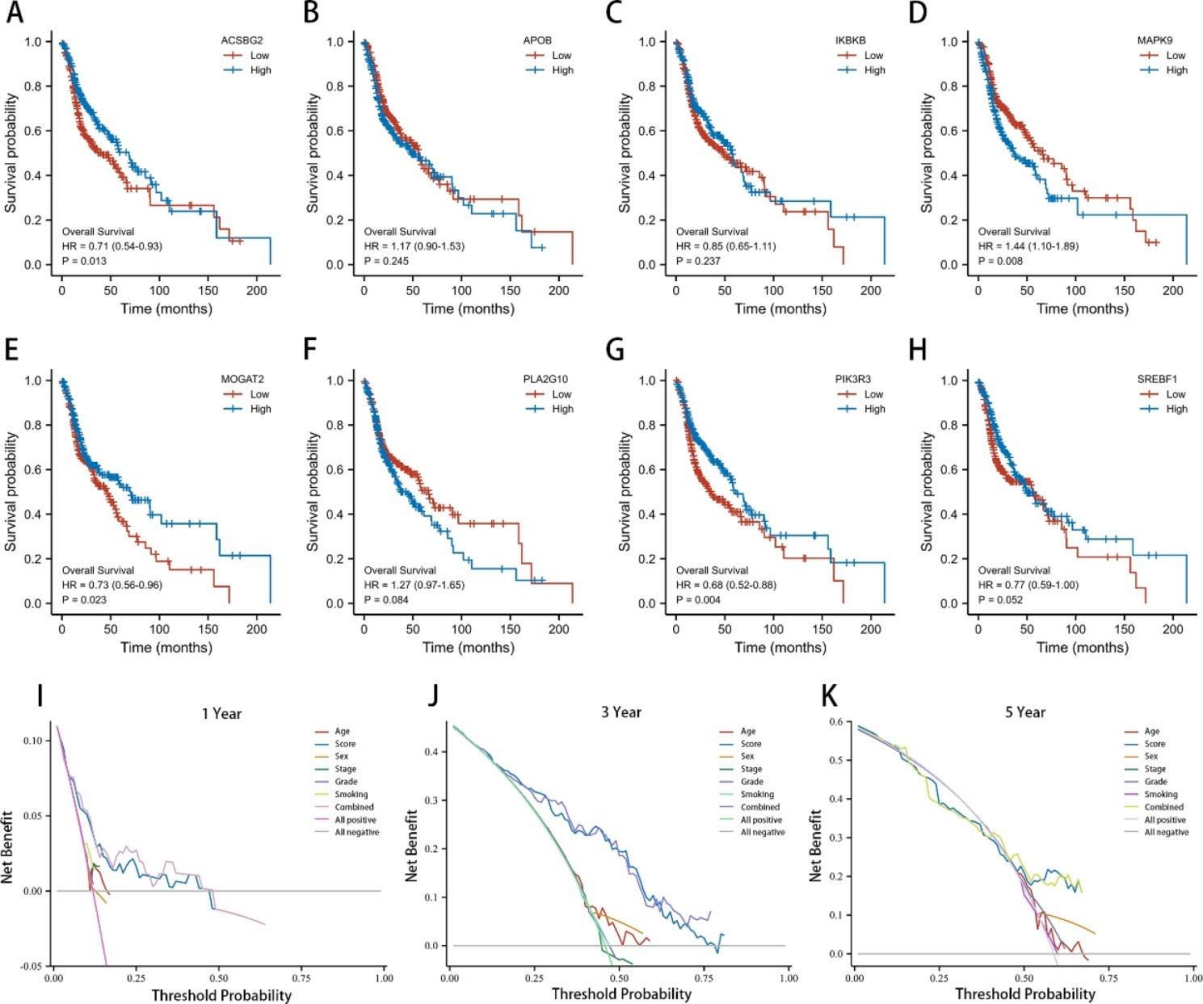
**The LMRS model had independent prognostic value when compared with single genes and other clinical characteristics. A–H**, Overall survival curve of the low- and high-expression subgroups for LMRS factors: ACSBG2, APOB, IKBKB, MAPK9, MOGAT2, PLA2G10, PIK3R3, and SREBF1. **I–K**, Decision curve analysis (DCA) for drug response prediction for HNSCCs with and without the LMRS model at 1, 3, and 5 years

In addition, MAPK9, MOGAT2, and PIK3R3 in the LMRS model exhibited significant survival differences in survival analysis among all TCGA-HNSCC patients with a p value < 0.05 (Fig. 4A–H). Moreover, low expression of MOGAT2 and PIK3R3 correlated with worse survival outcomes (Fig. 4E and G). These findings not only indicate that a single gene in the LMRS model could not serve as a diagnostic marker for HNSCCs but also support the theory that a higher LMRS score correlates with a worse survival outcome in HNSCCs. Combining these results, we infer that individuals with low LMRS may benefit more from chemotherapy or targeted drugs for the treatment of HNSCCs. In this LMRS model, the calculated weight of multiple genes was negative, and their relatively high expression was more likely to obtain a lower score, which is consistent with the prediction result. The DCA results demonstrated that, compared to other clinical characteristics, such as age, gender, clinical stage, pathological classification, and smoking status, the LMRS model, derived from eight lipid metabolism-related genes, had better evaluation efficiency. Notably, at three years, the LMRS model displayed significantly higher diagnostic decision benefits (Fig. 4I–K). Therefore, the LMRS model comprising eight lipid metabolism-related genes could have a similar evaluation effect to that of the 751 lipid-related gene classification model and outperform the single gene model in prognosis and drug response prediction for HNSCC.

### The LMRS model predicts the response to immune therapy and infiltration of immune cells

**Fig. 5:**
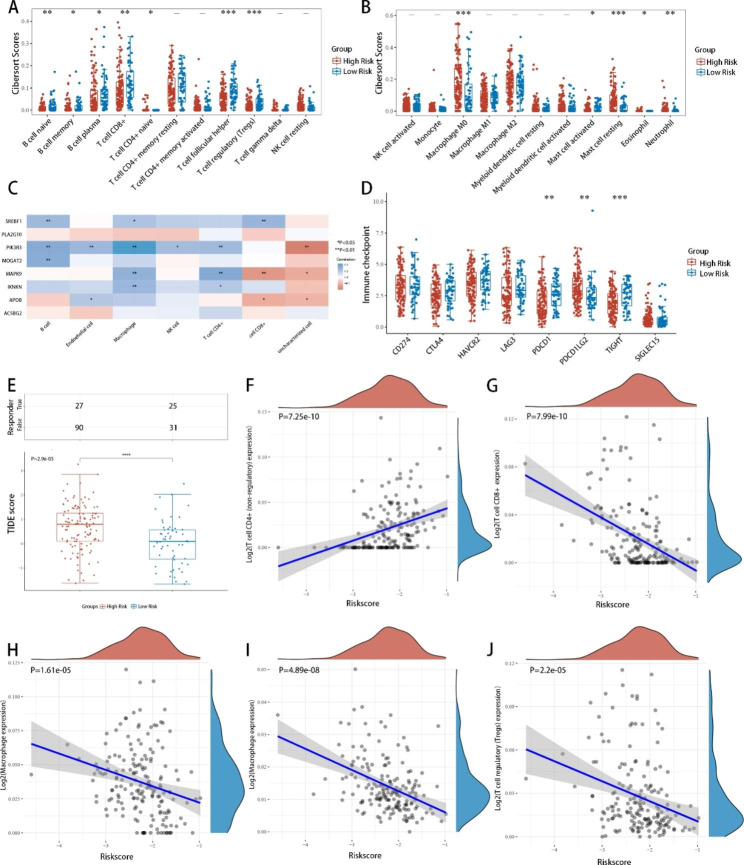
**Immune function-related evaluation of the eight-gene LMRS model. A–B**, Variation in immune cell infiltration between groups with high and low LMRS. **C**, Correlation between each immune cell infiltration and the genes in the LMRS model. **D**, Correlation between immune checkpoint marker expression levels and genes in the LMRS model. **E**, TIDE analysis of LMRS-high and -low groups. **F–J**, LMRS and correlation with CD4+, CD8+, M2 macrophages, NK cells, and T regulatory cells

Further investigation was conducted to evaluate the efficacy of the LMRS model in predicting immune-related functions. Our findings revealed a robust predictive effect of the LMRS model on immune cell infiltration that was particularly evident in T cells. CIBERSORT analysis results indicated that the LMRS-Low group had higher percentages of T-helper and T-reg cells and lower M0 macrophage infiltration (Fig. 5A, B). It is worth noting that not all genes in the LMRS model corresponded to immune cell infiltration levels (Fig. 5C). Therefore, the utilization of the eight-gene model for daily assessment is indispensable. The expression levels of ICB-related genes were examined, and the LMRS-Low group exhibited upregulation of PDCD1 and TIGIT and downregulation of PDCD1LG2. (Fig. 5D). Consistent with these findings, TIDE scores [23] demonstrated a better therapeutic response to immune checkpoint inhibitors and longer posttreatment survival in the LMRS-Low group with a p value < 0.05 (Fig. 5E). Conversely, the LMRS-High group was associated with elevated CD4 + T cells and reduced CTLs, M2 macrophages, NK cells, and Treg cells, with p values < 0.05 (Fig. 5F–J), suggesting that the LMRS-High groups were less likely to benefit from immune therapy. Our findings support the proficiency of the LMRS model in predicting the effectiveness of systemic therapy, including ICB, and the prognosis of HNSCCs.

### The efficacy of the LMRS model was validated in external HNSCC datasets

**Fig. 6:**
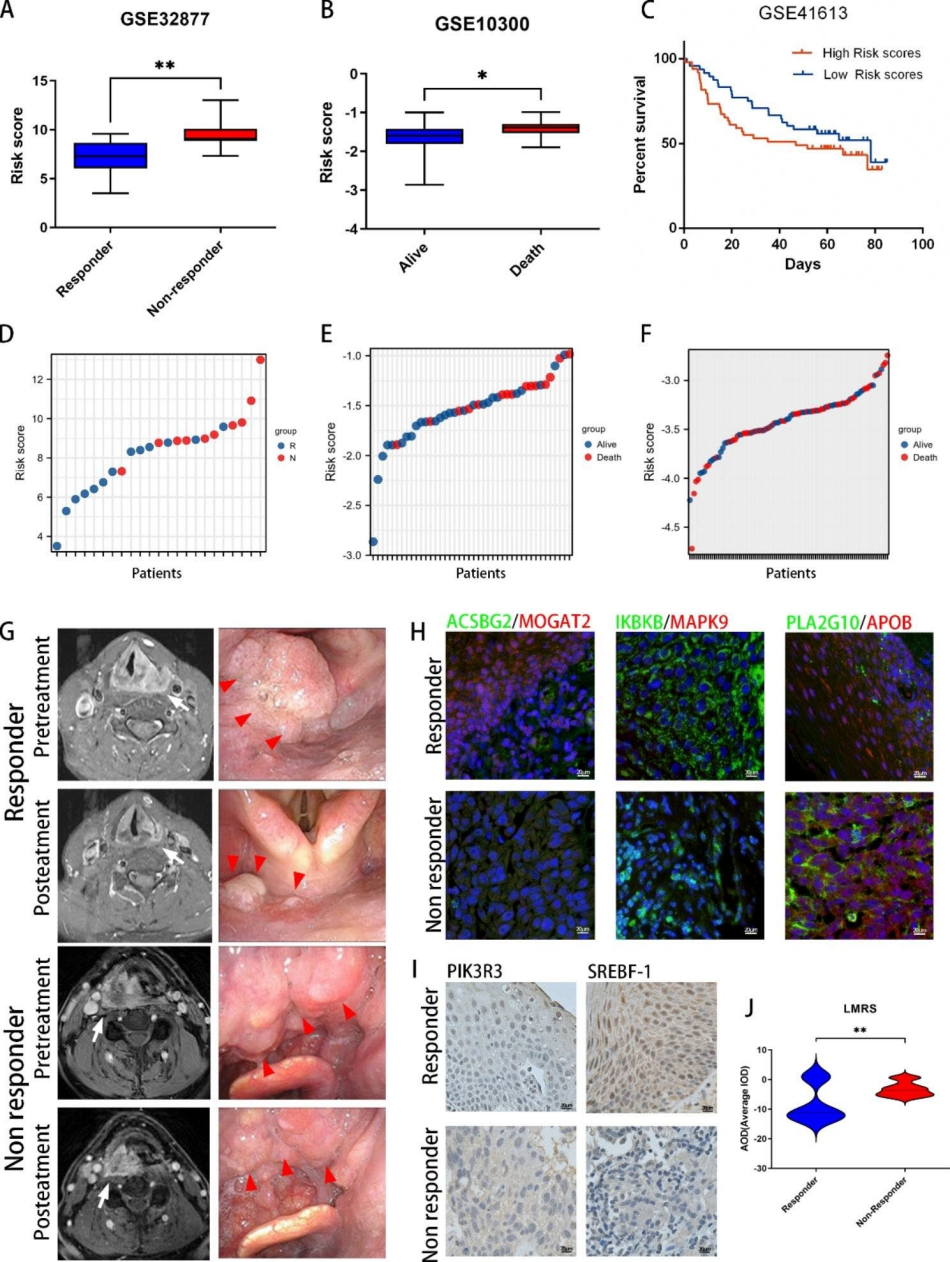
**Model verification and functional analysis of HNSCC data in other datasets. A**, LMRS scores of the GSE32877 HNSCC dataset between responder and nonresponder patients. **B**, LMRS scores of the GSE10300 HNSCC dataset between surviving and nonsurviving patients. **C**, Survival curve of the low- and high-LMRS groups in the GSE41613 HNSCC dataset. **D**, LMRS and chemotherapy response status of each patient in GSE32877. **E**, LMRS and survival status of each patient in GSE10300. **F**, LMRS and survival status of each patient in GSE41613. **G**, MRI and electronic laryngoscopy photographs of representative responder and nonresponder HNSCC patients. **H**, IF staining of ACSBG2 (green)/MOGAT2 (red), IKK2 (green)/MAPK9 (red) and PLA2G10 (green)/APOB (red) in the two groups. **I**, IHC staining of SREBF1/PIK3R3 in the two groups. **J**, Average IOD for the LMRS scores in responder and nonresponder groups

A limited number of datasets in GEO provide both treatment information and prognostic data for HNSCC. This study involved three HNSCC datasets from GEO, including a study examining the effectiveness of chemotherapy in HNSCC patients (GSE32877, n = 23; Fig. 6A), a study examining the survival and prognosis of HNSCC patients (GSE10300, n = 42; Fig. 6B), and a study examining the chemotherapy and survival information (GSE41613, n = 97; Fig. 6C). Gene expression data were retrieved from the GEO database, and corresponding LMRS scores were evaluated for each case using the formula of the model (Fig. 6D–F). Our findings revealed that patients who responded well to chemotherapy had a lower LMRS score (p value = 0.0032; n = 13), and those with prolonged survival exhibited a lower LMRS score (p value = 0.0151; n = 27). Moreover, the results of GSE41613 are consistent with the overall trend that patients with a higher LMRS score had a worse prognosis.

### The LMRS model predicts HNSCC chemotherapy response in clinical samples

In addition to the GEO datasets, we also verified the efficiency of the LMRS model in HNSCC specimens (n = 30). After the rounds of induction chemotherapy, we divided the patients into two subgroups. Those who exhibited a reduction greater than 50% fulfilled the responder status (Fig. 6G), whereas those with a decrease below 50% assumed the nonresponder status. Validation was performed using MRI and electronic laryngoscopy. Immunohistochemical (IHC) and immunofluorescence (IF) staining of the eight LMRS proteins corroborated the aforementioned bioinformatic findings in both cohorts (Fig. 6H–J). In the responder subgroup, the LMRS score was significantly lower than that in the nonresponder cohort (p value = 0.0097) (Fig. 6J). These findings further demonstrate the suitability of the LMRS model as an investigative tool to assess HNSCC treatment outcomes and drug response.

## Discussion

The significant contribution of glucose and glutamine metabolism reprogramming has been well established in a variety of cancers [29–32]. Recently, lipid metabolism has emerged as a factor related to treatment resistance in cancer cells, as it facilitates energy supply, the modulation of membrane fluidity, and the activation of signaling pathways [17]. Consequently, understanding the underlying mechanisms of lipid metabolism can help identify novel therapeutic targets for drug resistance in HNSCC. Despite the importance of metabolic conditions of HNSCC in relation to treatment response, few studies have investigated the potential links between chemotherapy resistance and lipid metabolism reprogramming. Therefore, an integrated analysis of predictive models for systematic therapy in HNSCCs is of significant value. In this study, we utilized CRISPR/Cas9 genome-wide library drug screening to identify key differentially expressed genes affecting chemotherapy resistance. In addition to DNA damage repair and traditional drug resistance pathways, our analysis revealed the crucial role of lipid metabolism in therapeutic resistance, as supported by functional enrichment analysis. Recent research has revealed the significance of lipid metabolism reprogramming in HNSCCs [17] and has indicated that it could promote tumor proliferation and metastasis [33–35], further underscoring the importance of lipid metabolism reprogramming. Our study demonstrated that lipid metabolism, particularly in chemotherapy-treated HNSCC patients, has a significant impact on survival.

In conjunction with TCGA database analysis, our findings suggest that lipid metabolism-related genes serve as valuable biomarkers for drug response in patients with HNSCCs. Generally, we identified 751 lipid metabolism-related genes that effectively divide HNSCC patients who received chemotherapy or targeted therapy into two subgroups, with each category exhibiting different clinical characteristics. Specifically, the C2 group had superior survival rates compared to the C1 group. However, the application of next-generation sequencing as a diagnostic tool for therapy decisions poses a significant challenge in identifying critical genes 31; this would be difficult to realize in daily clinical practice. Subsequently, combined with the results of sgRNA library screening and bioinformatics analysis, we reduced the number of genes to eight lipid metabolism-related genes. Remarkably, the subsequent analysis found that the established LMRS model performed well in evaluating the survival outcome and drug response of HNSCCs and performed with similar efficacy to that of the 751 lipid metabolism gene model. Although not every gene was differentially expressed in the TCGA-HNSCC database, the LMRS model highlighted frequently altered genes in HNSCCs, such as IKBKB [32], MAPK9 [38], and APOB [34]. Moreover, when combined with age, gender, tumor stage, pathological classification, and smoking status, the LMRS model effectively predicted short-term and medium-term diagnostic values, surpassing other clinical parameters, such as age and gender. However, additional research will be required to observe its long-term diagnostic potential. Our study demonstrates the significance of LMRS in predicting drug responses in HNSCC treatment.

It is worth noting that IKBKB [24], APOB [35] and MAPK9 [41] are not only vital cholesterol metabolism regulation genes but are also often upregulated in cancer, which implies that the use of cholesterol-lowering drugs in HNSCC patients with the overexpression of these genes may be helpful. The remaining genes, such as ACSBG2 [42], MOGAT2 [43], SREBF1 [44], PIK3R3 [45] and PLA2G10 [46], are involved in fatty acid metabolism; however, few studies have investigated their roles in the drug response to HNSCC chemotherapy. Our results in the present study were similar, since two different lipid metabolism-regulating drugs affect different LMRS genes.

Additionally, we found that immune cell infiltration and ICB-related gene expression were associated with the LMRS score. As the score increases, CD4 + T cells increase, and CTLs, M2 macrophages, NK cells, and Treg cells decrease. Moreover, the LMRS-High group was associated with the downregulated expression of immune checkpoints, such as TIGIT and PDCD1. TIGIT, a CD28 family member [42], inhibits T-cell activation by binding to CD155 [48]. TIGIT inhibition improved CD8 T-cell activation and prognosis in gastric cancer [44]. This association implies that both innate and acquired immunity are suppressed in the LMRS-High group. This immunological status in the LMRS-High group may explain why the LMRS-High group is associated with a worse prognosis. Many challenges are still unsolved for cancer immunotherapies in HNSCC [45]. Our LMRS model not only helps in identifying predictive biomarkers but also may enhance the prediction of the clinical response to HNSCC immunotherapy. More importantly, our results in the present study show the importance of lipid metabolism in the tumor microenvironment and the therapeutic potential of lipid-lowering drugs or lipid supplementation during the chemotherapeutic treatment of HNSCC.

The diagnostic accuracy of the LMRS model was validated through the examination of clinical HNSCC samples and the GEO database. Given the potential for frequent monitoring and reduced costs, a diagnostic kit incorporating the eight identified genes could potentially assist in drug response assessments for patients with HNSCC, aiding in tailored treatment selection. For patients in the LMRS-High group who are not sensitive to drug treatment, alternative treatment strategies or therapeutic sensitizer should be offered [46, 47], such as dietary intervention, or blocking downstream lipid metabolism. Considering that the expression levels of risk factors are higher, and the expression levels of protective factors might be lower in the LMRS-High group, lipid-lowering drugs or drugs targeting risk factor genes would be a potential choice. For sensitive patients in the LMRS-Low group, successful introduction of drug therapy is critical for improving overall patient survival rates, achieving locoregional control, decreasing cancer fatalities [48], and downstaging tumors into a resectable size. Specific lipid supplementation may also be helpful, although more specific and in-depth studies are needed to clarify its effects. Postradiotherapy or postsurgery, chemotherapy and immunotherapy may be useful as potential adjuvant treatments based on the individual patient’s needs. Importantly, reducing tumor size can facilitate inoperable HNSCC cases to become operable and further aid in preserving language, respiratory, and swallowing functions.

## Conclusion

In conclusion, this study employed a combination of CRISPR/Cas9 library screening and bioinformatics database mining to investigate lipid metabolism genes associated with drug response in HNSCCs. Utilizing the data obtained, an LMRS model capable of evaluating the clinical outcomes and drug responses of HNSCCs was developed, facilitating the recognition of individuals who are unlikely to respond to drug therapy. This not only saves time and costs for patients but also provides a foundation for the development of novel drugs. Furthermore, the LMRS model showed potential as a predictive tool for ICB therapy for HNSCCs. Our results imply that targeting these genes may hold potential as a viable therapeutic strategy for HNSCC treatment. Additional research is needed to assess the long-term value and effectiveness of the LMRS model under different nutritional statuses.

### Electronic supplementary material

Below is the link to the electronic supplementary material.

Supplementary Material 1

## Data Availability

The data generated in this study are available within the article and its supplementary data files. Clinical information, TCGA RNA-seq data, and probe annotation files were retrieved from the TCGA HNSCC dataset (https://portal.gdc.com). Expression profile data analyzed in this study were obtained from Gene Expression Omnibus (GEO) at GSE32877, GSE10300, and GSE41613. The sequence data generated in this study are available in NCBI, BioProject with ID: PRJNA948554.

## Abbreviations

ACSBG2: Acyl CoA synthetase bubblegum family member 2
APOB: Apolipoprotein
BPs: Biological processes
CCs: Cellular components
CTL: Cytotoxic lymphocyte
EGFR: Epidermal growth factor receptor
HNSCC: Head and neck squamous cell cancer
HPV: Human Papilloma virus
IKBKB: Inhibitor of kappa light polypeptide gene enhancer in B-cells, kinase epsilon, B
IF: Immunofluorescence
IHC: Immunohistochemical
LASSO: Least absolute shrinkage and selection operator
LMRS: Lipid metabolism related score
MAPK9: Mitogen-Activated Protein Kinase 9
MFs: Molecular functions
MOGAT2: Monoacylglycerol-O-acyltransferase 2
MRI: Magnetic resonance imaging
OS: Overall Survival
PD-1/PD-L1: Programmed Cell Death 1/programmed Cell Death 1 Ligand 1
PIK3R3: Phosphoinositide-3-Kinase Regulatory Subunit 3
PLA2G10: Phospholipase A2 Group X
SREBF1: Sterol Regulatory Element Binding Transcription Factor 1
TCGA: The Cancer Genome Atlas
TIDE: Tumor immune dysfunction and exclusion
