# Supplementary material for: Enhancing prognostic accuracy in head and neck squamous cell carcinoma chemotherapy via a lipid metabolism-related clustered polygenic model
Source: Cancer Cell Int. 2023 Aug 11;23:164. doi: 10.1186/s12935-023-03014-5 (PMC10422777; doi:10.1186/s12935-023-03014-5)
Supplement: Supplementary file 1 — Supplementary Material 1 [file 12935_2023_3014_MOESM1_ESM.docx]

**Supplementary material**

**Table S1. IC20 and IC50 of cisplatin and 5-FU in Fadu and Detroit 562**

|  | **Fadu** | | | | **Detroit 562** | | | |
| --- | --- | --- | --- | --- | --- | --- | --- | --- |
|  | **Cisplatin** | | **5-FU** | | **Cisplatin** | | **5-FU** | |
| **IC20** | 2.54 | ±0.99 | 1.63 | ±0.39 | 1.25 | ±0.55 | 0.07 | ±0.64 |
| **IC50** | 8.22 | ±2.30 | 6.08 | ±0.81 | 17.35 | ±2.98 | 2.91 | ±1.62 |

（Unit: μg/ml）

**Table S2. Characteristics of TCGA HNSCCs patient’s cohort (received chemo- or targeted therapy)**

| **Characteristic** | **LMRS-High** | **LMRS-Low** | **P Value** |
| --- | --- | --- | --- |
| **Number** | **117** | **56** |  |
| **Sex, n (%)** |  |  | **0.306** |
| Female | 26 (15%) | 8 (4.6%) |  |
| Male | 91 (52.6%) | 48 (27.7%) |  |
| **Race, n (%)** |  |  | **0.420** |
| American Indian | 1 (0.6%) | 0 (0%) |  |
| Asian | 5 (2.9%) | 0 (0%) |  |
| Black | 12 (7.1%) | 7 (4.1%) |  |
| White | 96 (56.5%) | 49 (28.8%) |  |
| **T, n (%)** |  |  | **0.707** |
| T1 | 4 (2.3%) | 3 (1.7%) |  |
| T2 | 26 (15%) | 15 (8.7%) |  |
| T3 | 37 (21.4%) | 14 (8.1%) |  |
| T4 | 50 (28.9%) | 24 (13.9%) |  |
| **N, n (%)** |  |  | **0.001*** |
| N0 | 44 (26%) | 8 (4.7%) |  |
| N1 | 14 (8.3%) | 11 (6.5%) |  |
| N2 | 54 (32%) | 33 (19.5%) |  |
| N3 | 1 (0.6%) | 4 (2.4%) |  |
| **M, n (%)** |  |  | **1.000** |
| M0 | 112 (67.1%) | 53 (31.7%) |  |
| M1 | 2 (1.2%) | 0 (0%) |  |
| **Stage, n (%)** |  |  | **0.601** |
| I | 2 (1.2%) | 0 (0%) |  |
| II | 7 (4%) | 1 (0.6%) |  |
| III | 14 (8.1%) | 7 (4%) |  |
| IV | 94 (54.3%) | 48 (27.7%) |  |
| **Grade, n (%)** |  |  | **0.769** |
| G1 | 5 (3.1%) | 3 (1.9%) |  |
| G2 | 77 (48.4%) | 32 (20.1%) |  |
| G3 | 27 (17%) | 13 (8.2%) |  |
| G4 | 1 (0.6%) | 1 (0.6%) |  |
| **Smoking, n (%)** |  |  | **0.431** |
| No | 26 (15.1%) | 16 (9.3%) |  |
| Yes | 91 (52.9%) | 39 (22.7%) |  |
| **Radiation, n (%)** |  |  | **0.368** |
| NO | 3 (4.5%) | 3 (4.5%) |  |
| YES | 43 (64.2%) | 18 (26.9%) |  |
| **Neoadjuvant, n (%)** |  |  | **0.596** |
| No | 115 (66.5%) | 54 (31.2%) |  |
| Yes | 2 (1.2%) | 2 (1.2%) |  |
| **Therapy, n (%)** |  |  | **0.103** |
| Chemotherapy | 109 (67.7%) | 50 (31.1%) |  |
| Immunotherapy | 0 (0%) | 2 (1.2%) |  |
| **Alcohol, n (%)** |  |  | **0.480** |
| NO | 26 (15%) | 9 (5.2%) |  |
| YES | 90 (52%) | 46 (26.6%) |  |
| Not Available | 1 (0.6%) | 1 (0.6%) |  |

**
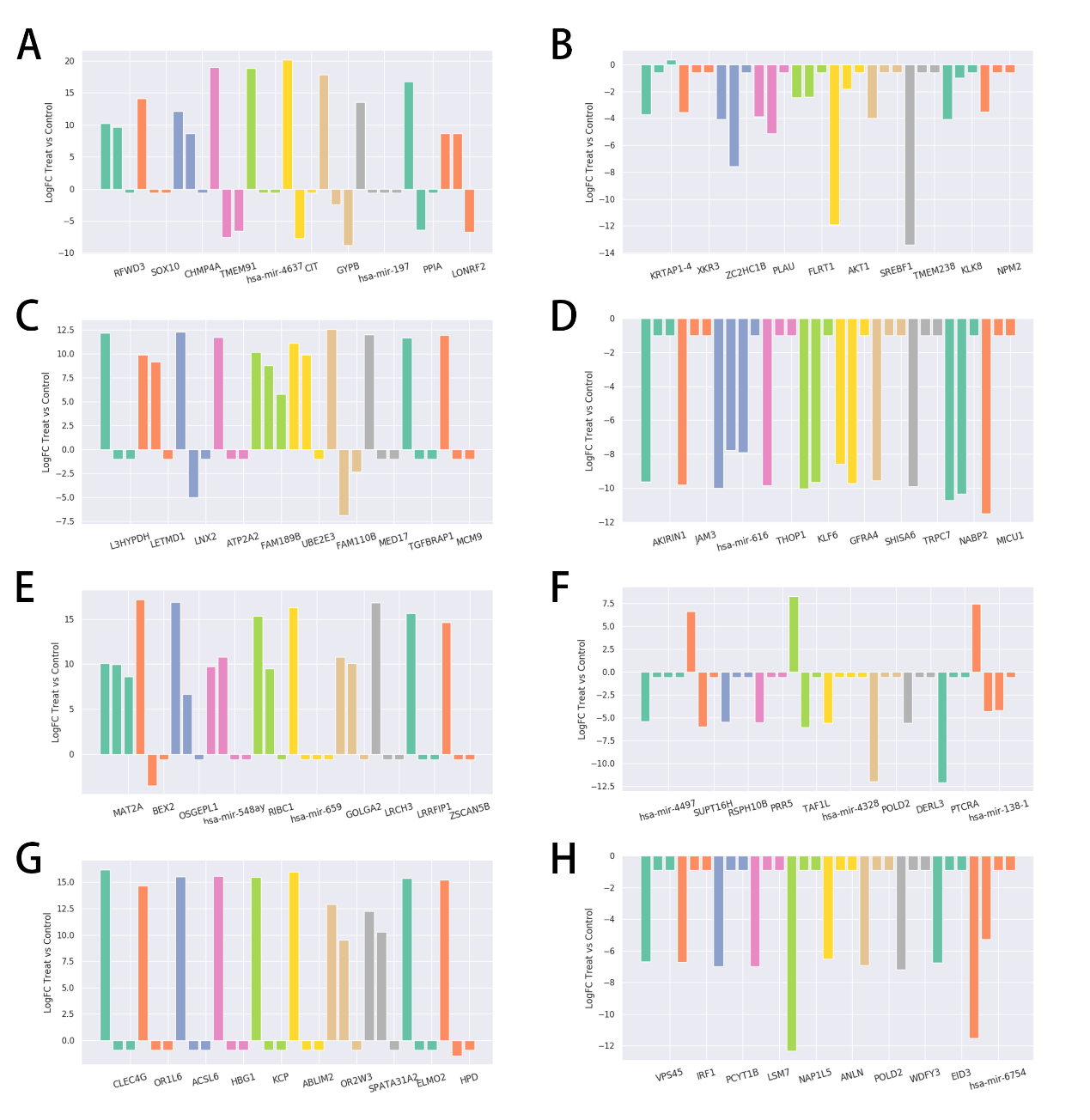
**

**Figure S1. Top 10 DEGs. A**, Top 10 positive differentially expressed sgRNA between cisplatin-treated and control groups in Fadu. **B**, Top 10 negative differentially expressed sgRNA between cisplatin-treated and control groups in Fadu. **C**, Top 10 positive differentially expressed sgRNA between 5-FU-treated and control groups in Fadu. **D**, Top 10 negative differentially expressed sgRNA between 5-FU-treated and control groups in Fadu. **E**, Top 10 positive differentially expressed sgRNA between cisplatin-treated and control groups in Detroit-562. **F**, Top 10 negative differentially expressed sgRNA between cisplatin-treated and control groups in Detroit-562. **G**, Top 10 positive differentially expressed sgRNA between 5-FU-treated and control groups in Detroit-562. **H**, Top 10 negative differentially expressed sgRNA between 5-FU-treated and control groups in Detroit-562.

**
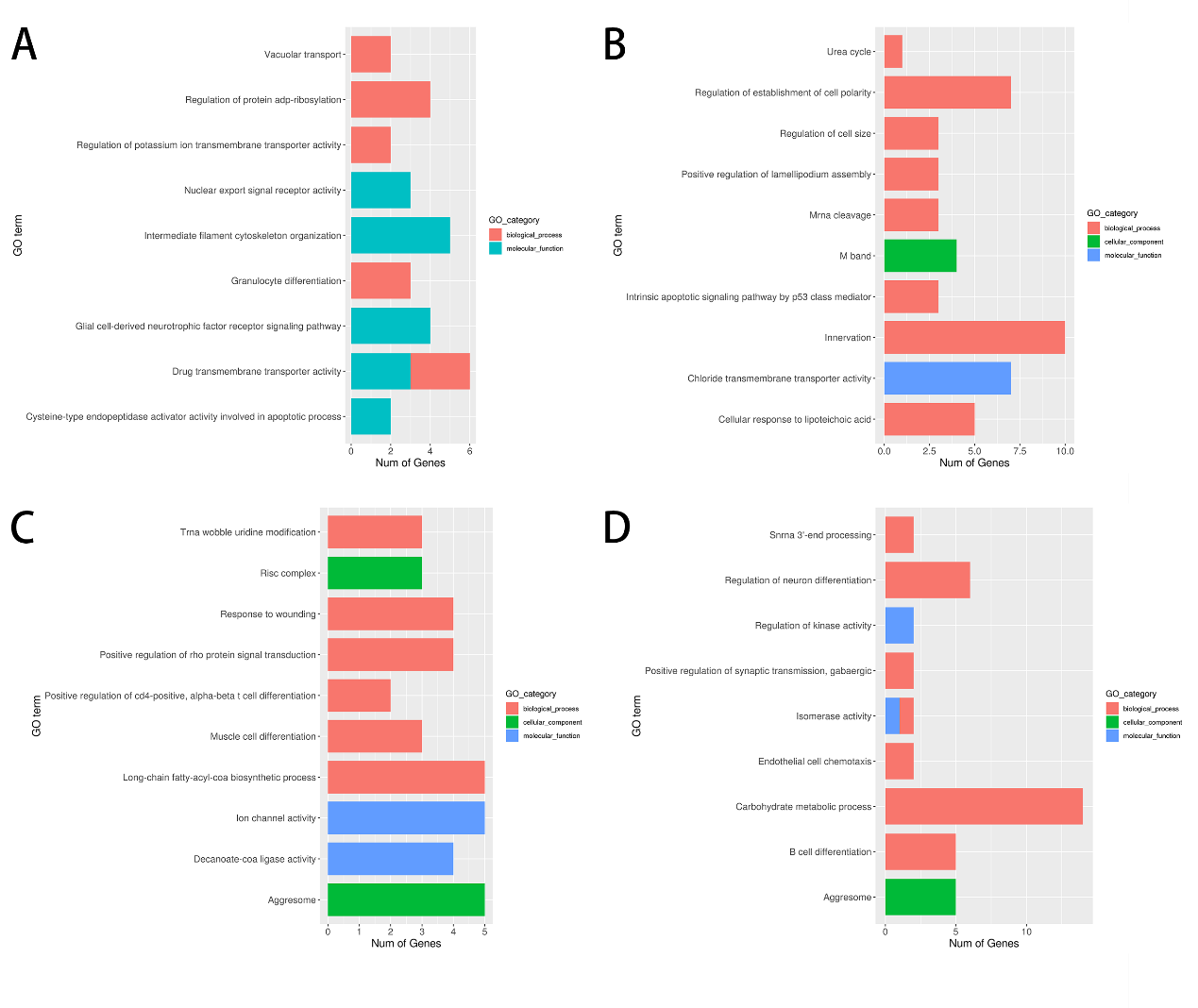
**

**Figure S2. GO analysis between drug-treated and control groups. A**, GO analysis results between cisplatin and control groups in Fadu. **B**, GO analysis results between 5-FU and control groups in Fadu. **C**, GO analysis results between cisplatin and control groups in Detroit-562. **D**, GO analysis results between 5-FU and control groups in Detroit-562.


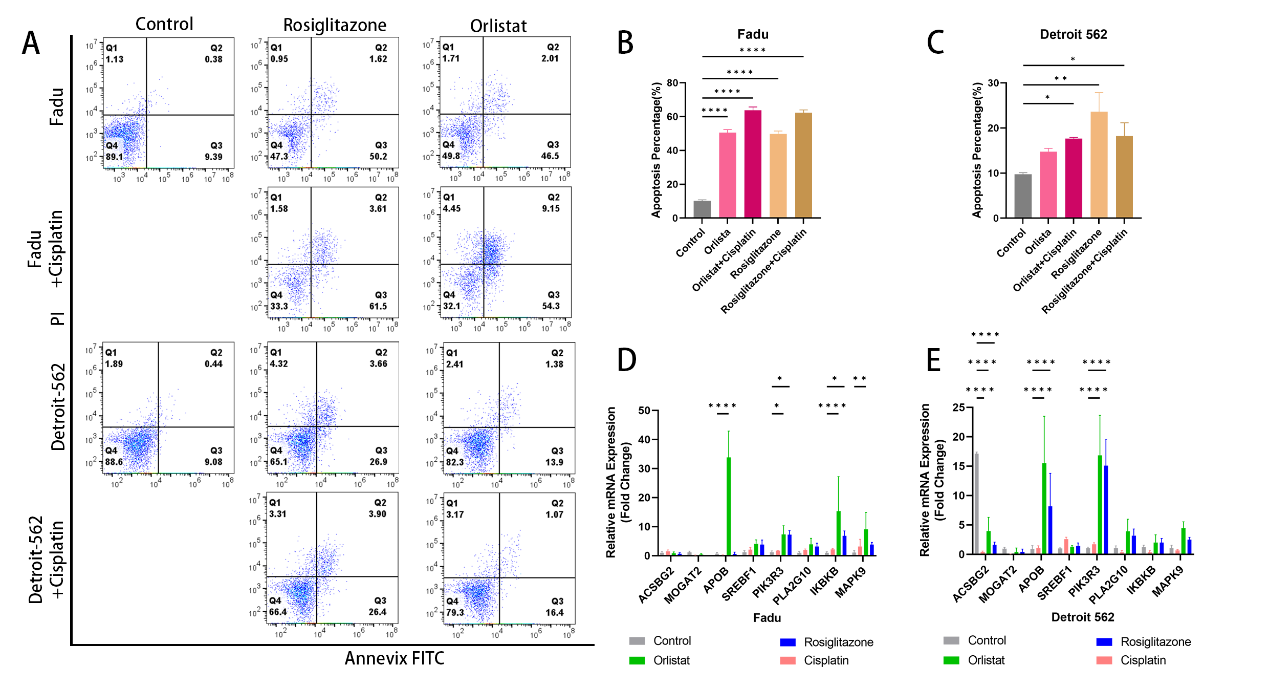
 **Figure S3. Effects of lipid metabolism regulation drugs in HNSCC cell lines. A,** Annexin V- PI assays of lipid metabolism drugs in HNSCC cell lines. **B,** Apoptosis percentage of lipid regulation drugs in Fadu. **C,** Apoptosis percentage of lipid regulation drugs in Detroit 562. **D,** Relative expression of LMRS model genes under the treatment of lipid metabolism drugs with cisplatin in Fadu. **E,** Relative expression of LMRS model genes under the treatment of lipid metabolism drugs with cisplatin in Detroit 562.

**
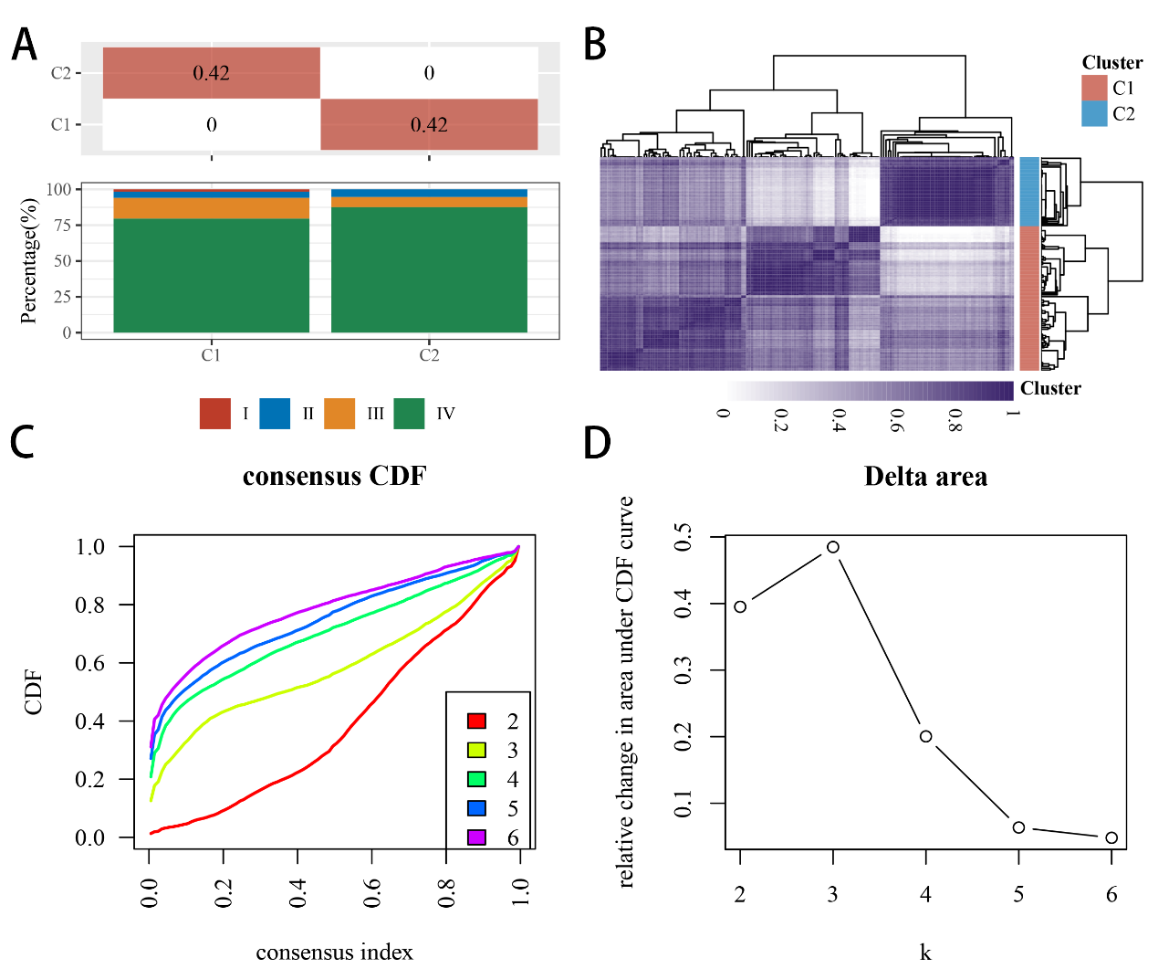
**

**Figure S4. Consensus clustering for the different lipid metabolism subgroups.** **A**, Tumor stages in C1 and C2 groups. **B**, Heatmap consistency of clustering results. Samples are represented by rows and columns, and subgroups are represented by various colors. **C**, CDF curve. **D**, CDF delta area curve.
